# The relationship between the severity of erectile dysfunction and the risk of co-occurrence with premature ejaculation: a cross-sectional study

**DOI:** 10.3389/fpubh.2026.1956425

**Published:** 2026-09-10

**Authors:** Yong Ouyang, Shi-An Hu, Jie-Dong Zhou, Si-Si Huang, Min Liu

**Affiliations:** 1 Department of Reproductive Medicine, The First Affiliated Hospital of Gannan Medical University, Ganzhou, Jiangxi, China; 2 The First Affiliated Hospital of Gannan Medical University, Ganzhou, China; 3 First School of Clinical Medicine, Gannan Medical University, Ganzhou, China

**Keywords:** comorbidity, cross-sectional study, erectile dysfunction, premature ejaculation, public health, severity

## Abstract

**Purpose:**

This study aimed to investigate the directional relationship and distributional differences between erectile dysfunction (ED) and premature ejaculation (PE) in clinical patients, and to explore whether ED severity predicts the risk of PE comorbidity.

**Methods:**

A cross-sectional, single-center study enrolled 230 men aged 22–45 years with sexual dysfunction (January 2023 – December 2024). ED and PE severity were assessed using the IIEF-5 and CIPE. Patients were classified as PE only (*n* = 120), ED only (*n* = 72), or comorbid ED-PE (*n* = 38). The McNemar test was used to evaluate asymmetry in comorbidity distribution, and comparisons were made across ED severity grades.

**Results:**

The prevalence of PE (68.7%) was higher than that of ED (47.8%). There was significant asymmetry: PE alone (*n* = 120) was more common than ED alone (*n* = 72); (*P* = 0.0007). ED severity showed a unidirectional association with PE risk; comorbidity rates increased from mild to moderate ED (22.2% → 42.9%) but plateaued thereafter (severe ED 43.3%). Severe ED had a higher PE risk than mild ED (RR = 1.95, *P* < 0.05). PE severity did not affect ED prevalence.

**Conclusion:**

This cross-sectional study suggests an asymmetric association pattern between ED severity and PE risk, most evident during the mild-to-moderate progression. Because of the cross-sectional design, these findings are preliminary and should be interpreted as associations rather than causal relationships. Prospective studies are needed to determine whether early ED progression is a critical risk window for PE. The mild-to-moderate ED group, representing a larger target population with the most pronounced risk escalation, warrants further investigation in future studies.

## 1 Introduction

Erectile dysfunction (ED) and premature ejaculation (PE) are the two most common sexual dysfunctions in adult men worldwide. They impair patients' sexual satisfaction and negatively affect their mental health, self-confidence, and partner relationships (1).

ED is defined as a persistent or recurrent inability to achieve or maintain an erection sufficient for satisfactory sexual intercourse. Its prevalence rises with age (2, 3): for example, approximately 20.1% in men aged 20–29, increasing sharply to about 70% in those aged 60–69 (4). Etiology is complex and involves multiple risk factors, including chronic physical conditions (e.g., diabetes, hypertension, cardiovascular disease, obesity), unhealthy lifestyle habits (e.g., smoking), and psychological/psychiatric issues (e.g., depression, anxiety) (5, 6).

PE is characterized by poor ejaculatory control, typically occurring within a very short time after vaginal penetration (often defined as <1 min), leading to significant personal distress or interpersonal difficulty. Based on onset, PE can be lifelong (since first sexual encounter) or acquired (developing later) (7). Global prevalence of PE ranges from 5 to 30% (8), with higher rates in the Asia-Pacific region; reports from some countries indicate rates between 16 and 30% (9, 10).

Previous studies have shown a high comorbidity rate between ED and PE (11, 12). Epidemiological data indicate that PE prevalence is significantly higher in ED patients than in the general population, and vice versa. This comorbidity is thought to result from complex interactions among psychological, physiological, and neurobiological factors (13). For example, anxiety about maintaining an erection (performance anxiety) may cause ED patients to ejaculate prematurely, leading to secondary PE. Conversely, chronic PE may cause sexual frustration and reduced self-confidence, which then impair erectile function (1, 14).

Although the comorbidity between ED and PE is widely recognized, the direction of the association between the two remains controversial. Some studies suggest a bidirectional relationship between ED and PE, meaning that PE may also increase the risk of developing ED. However, from a pathophysiological perspective, ED-related endothelial dysfunction, chronic inflammation, and compensatory behavioral changes triggered by anxiety may more directly affect ejaculatory control. Furthermore, in clinical practice, the rate of self-reported comorbidity of PE among ED patients is low, suggesting that the pathway by which ED progresses to PE may not be fully recognized in clinical settings. Based on these considerations, this study focuses on exploring the association between the severity of ED and the risk of PE comorbidity, aiming to describe the distribution characteristics of both conditions across severity levels rather than to establish a causal relationship. This study hypothesizes that: (1) the distribution of comorbidity between ED and PE is asymmetric; and (2) the severity of ED is associated with the risk of PE comorbidity. Given the cross-sectional nature of this study, these hypotheses are intended to describe association patterns rather than to establish causal relationships. We aim to describe the comorbidity patterns between ED and PE across different severity grades, thereby providing foundational data for future hypothesis-driven research.

From a public health perspective, clarifying this association is crucial for developing population-level screening guidelines and targeted health education interventions for young and middle-aged men—a demographic that carries a significant yet often neglected burden of sexual health issues.

## 2 Materials and methods

### 2.1 Study design and ethics

This retrospective cross-sectional study was approved by the Ethics Committee of the First Affiliated Hospital of Gannan Medical University (Ethics No.: LLSC-2025-214) and registered with the Thailand Clinical Trials Registry (TCTR20250526008). All procedures performed in this study were in accordance with the ethical standards of the institutional research committee and with the 1964 Declaration of Helsinki and its later amendments.

The requirement for informed consent was waived by the Ethics Committee because of the retrospective nature of the study and the use of anonymized data collected during routine clinical practice. All data were de-identified according to hospital regulations. The study was designed and reported in accordance with the STROBE (Strengthening the Reporting of Observational Studies in Epidemiology) guideline.

### 2.2 Study population

Between January 2023 and December 2024, this study included 230 adult male patients presenting with erectile dysfunction and/or premature ejaculation at the outpatient clinic of the Department of Reproductive Medicine, The First Affiliated Hospital of Gannan Medical University.

#### 2.2.1 Inclusion criteria

Eligible participants were men aged 22–45 years with a stable heterosexual partner and regular sexual activity for at least 6 months. Sexual activity frequency was defined as the average number of sexual intercourse attempts per month over the preceding 6 months, assessed by patient self-report. ED was defined as an IIEF-5 score ≤ 21, with symptoms persisting for ≥ 3 months and at least one intercourse attempt within the past 4 weeks. PE was defined according to the International Society for Sexual Medicine (ISSM) criteria, including lifelong PE (intravaginal ejaculatory latency time [IELT] <1 min) or acquired PE (significant shortening of IELT), with symptoms lasting ≥ 3 months and at least one intercourse attempt within the past 4 weeks. All participants had confirmed ED and/or PE; no healthy control group was included, as the study aimed to explore comorbidity patterns rather than compare patients with the general population.

#### 2.2.2 Exclusion criteria

Patients were excluded if they had any of the following: severe cardiovascular disease, uncontrolled hypertension, severe hepatic or renal insufficiency, psychiatric or cognitive disorders, active malignancy, use of medications affecting sexual function within the past 3 months (e.g., certain antihypertensives or antidepressants), anatomical abnormalities of the reproductive organs, active genitourinary infection, history of pelvic surgery, alcohol or substance dependence, or current participation in other clinical studies. Additionally, individuals with no sexual activity in the preceding 6 months, incomplete questionnaire data, or a score of zero were excluded.

### 2.3 Research tools and data collection

The study utilized internationally recognized standardized scales to assess patients' sexual function:

International Index of Erectile Function Short Form (IIEF-5) (15): Used to assess the severity of erectile dysfunction. This scale consists of 5 questions, each scored on a 1–5 point scale, with a total score ranging from 5 to 25 points. A lower score indicates more severe ED. ED is typically classified as: no ED (22–25 points), mild ED (17–21 points), mild-to-moderate ED (12–16 points), moderate ED (8–11 points), and severe ED (5–7 points). For the purposes of the present analysis, mild ED and mild-to-moderate ED were combined into a single “mild ED” category to ensure sufficient sample size for subgroup analysis and to focus on the comparison between non-severe and severe ED stages. This modified classification is justified by the small sample size in the mild-to-moderate subgroup and is consistent with the analytical approach of previous cross-sectional studies in this field.

Chinese Index of Sexual Function for Patients with Premature Ejaculation (CIPE) (16): This quantitative tool consists of 5 questions, each scored on a 5-point Likert scale (1–5), with a total score ranging from 5 to 25. Lower scores indicate more severe PE. Employing the total score of CIPE-5, patients with PE were divided into three groups: mild (> 15 points), moderate (10–14 points), and severe (<9 points), consistent with the original validation study [Yuan et al., 2004]. For the present analysis, these categories were used as defined in the original study. The CIPE was used as the primary instrument for PE severity grading in this study.

Intravaginal Ejaculatory Latency Time (IELT) (17): IELT was defined as the time from vaginal penetration to ejaculation. **IELT** **<** **1 min was used as a diagnostic criterion for lifelong PE in accordance with the International Society for Sexual Medicine (ISSM) definition**. In this retrospective study, IELT was assessed through patient self-report during clinical interviews, as stopwatch timing was not systematically employed due to practical feasibility constraints in the outpatient clinical setting. Patients were asked to report their typical IELT over the preceding 4 weeks. All IELT data used for PE diagnosis were based on patient self-report. **IELT data were used for diagnostic classification purposes and were not included as primary analytical variables for statistical modeling**. Consistent with Lee et al., we acknowledge that self-reported and stopwatch-measured IELT may not be directly interchangeable; however, self-reported IELT is widely accepted in clinical practice for screening and diagnostic purposes.

### 2.4 Statistical analysis strategy

All collected data were analyzed using SPSS (Version 27.0). Descriptive statistics: The general demographic characteristics (e.g., age, weight, BMI, marital status) and clinical characteristics of the patients were described. Continuous variables are expressed as mean ± standard deviation (Mean ± SD), and categorical variables are expressed as frequency and percentage (*n*, %). Comparisons between groups were performed using the chi-square test or analysis of variance (ANOVA).

To explore the relationship between ED severity and the risk of PE comorbidity, we planned a multilevel analysis strategy in advance. The Cochran-Armitage trend test was used to assess the overall linear trend; given that the comorbidity rates in the moderate and severe groups were similar (which might violate the proportional odds assumption of ordered logistic regression), we supplemented the analysis with multinomial logistic regression (using mild ED as the reference group) to calculate odds ratios (OR) and their 95% confidence intervals. All statistical tests were two-sided, with a significance level (α) set at *P* < 0.05. It should be noted that the planned Spearman correlation analysis could not be performed due to missing data (*see Section 2.7*).

To further assess the independent association between ED severity and PE presence, we performed a multivariable binary logistic regression analysis with PE presence (yes/no) as the dependent variable and ED severity (mild, moderate, severe) as the independent variable, adjusted for age, BMI, and marital status (covariates available in the dataset). Results are presented as adjusted odds ratios (aOR) with 95% confidence intervals.

### 2.5 *Post-hoc* sample size estimation

This study is an exploratory cross-sectional survey, and the sample size was primarily determined based on feasibility. Using the expected difference in comorbidity rates (OR ≈ 2.0), α = 0.05, and β = 0.20 for *post-hoc* estimation, the current sample size (*N* = 230) has approximately 80% statistical power to detect an association of moderate effect size.

### 2.6 Sensitivity analysis

To assess the robustness of the main findings, we conducted two *post hoc* sensitivity analyses: (1) logistic regression with the IIEF-5 score as a continuous variable, with PE presence as a binary outcome (present vs. absent); and (2) repeating the primary analysis after excluding borderline cases (IIEF-5 = 21 or CIPE-5 = 8–9). The main conclusions remained stable in these analyses (see Supplementary Table S2).

### 2.7 Limitations of the data analysis

We acknowledge the following analytical limitations: (1) Spearman correlation analysis was unavailable due to missing data; (2) although we used multinomial logistic regression to avoid violating the proportional odds assumption of ordered logistic regression, this assumption was not formally tested; (3) multiple comparisons were adjusted using Bonferroni correction (corrected α = 0.025), which may increase the risk of Type II errors; (4) the severe ED subgroup was small (*n* = 30), limiting the precision of estimates. All results should be interpreted within this methodological context. Additionally, IELT measurements relied solely on patient self-report in this study, as stopwatch timing was not systematically implemented. Although self-reported IELT is widely accepted in clinical practice, the absence of objective timing may have introduced variability in IELT estimation.

## 3 Study results

### 3.1 General demographic characteristics of the study population

A total of 230 patients were included in this study. Based on diagnostic results, patients were divided into three groups: PE only (*n* = 120, 52.17%), ED only (*n* = 72, 31.30%), and comorbid ED-PE (*n* = 38, 16.52%).

Table 1 summarizes the demographic and clinical characteristics across the three groups. Although the mean age was slightly higher in the ED-only (35.8 ± 5.9 years) and comorbid groups (36.6 ± 6.1 years) compared with the PE-only group (33.6 ± 5.3 years), the differences were not statistically significant (*P* = 0.249). No significant differences were observed in weight, BMI, marital status, marriage duration, or frequency of sexual activity among the three groups (all *P* > 0.05; Table 1). Sexual activity frequency was similar across the three groups (PE-only: 1.63 ± 0.70; ED-only: 1.60 ± 0.70; comorbid: 1.22 ± 0.65 times/month; *P* = 0.125), indicating comparable baseline sexual activity levels despite differences in erectile function status. The lower frequency in the comorbid group, while not statistically significant, may reflect the combined impact of both ED and PE on sexual behavior. As expected by the grouping criteria, the PE-only group had significantly higher IIEF-5 scores (23.0 ± 2.5) compared with the ED-only (13.3 ± 4.5) and comorbid (10.1 ± 4.8) groups (*P* < 0.001), confirming the validity of the diagnostic classification (Table 1).

**Table 1:** General demographic and clinical characteristics of the study population (*N* = 230).

Variable	PE only (*n* = 120)	ED only (*n* = 72)	ED + PE (*n* = 38)	*P-*value
**Age (years)**	33.6 ± 5.3	35.8 ± 5.9	36.6 ± 6.1	0.249
**Weight (kg)**	60.8 ± 7.6	63.8 ± 9.3	66.2 ± 8.7	0.152
**BMI (kg/m** ^**2**^ **)**	21.5 ± 2.4	22.3 ± 2.9	23.0 ± 2.7	0.224
**Married (%)**	55.0	75.0	78.9	0.216
**Marriage duration (years)**	18.6 ± 11.7	19.6 ± 10.4	23.6 ± 8.6	0.290
**Sexual activity (times/month)**	1.63 ± 0.70	1.60 ± 0.70	1.22 ± 0.65	0.125
**IIEF-5 score**	23.0 ± 2.5	13.3 ± 4.5	10.1 ± 4.8	<0.001

Data are presented as mean ± SD for continuous variables and as percentage (%) for categorical variables. Continuous variables were compared using one-way ANOVA; categorical variables were compared using the chi-square test. Marital status and marriage duration were assessed among married participants. IIEF-5 scores are presented for descriptive purposes; the PE-only group had significantly higher IIEF-5 scores than the ED-only and comorbid groups by definition of the grouping criteria. No post-hoc pairwise comparisons were performed.

### 3.2 Distribution of comorbidities between ED and PE

Given that the two sample groups share the same pool of patients with comorbidities, this comparison does not employ independent-samples significance tests; instead, the McNemar test was used to assess asymmetry in the distribution of ED and PE, as the two diagnostic categories are not independent (i.e., patients with both conditions are counted in both groups). Inferential results are presented in Table 2. Statistical analysis revealed that ED and PE co-occurred in a subset of patients; among the 230 patients, 38 (16.5%) had both conditions, while 192 (83.5%) had only one of the two conditions. The proportion of patients with PE was 68.7% (158/230), higher than that of ED (47.8%, 110/230). The number of patients with PE alone (*n* = 120) was significantly larger than the number with ED alone (*n* = 72), indicating a marked asymmetry in the comorbidity pattern (*P* = 0.0007, McNemar's test).

**Table 2:** Distribution of patients with PE and ED by single condition and comorbidity (McNemar test frequency table).

Condition	ED(+)	ED(-)	Total
**PE(+)**	38	120	158
**PE(–)**	72	0	72
**Total**	110	120	230

McNemar test indicated that the marginal probabilities for the two outcomes were not equal (χ^2^ = 11.505, *P* = 0.0007; exact *P* = 0.00066). The odds ratio for the difference between discordant pairs was b/c = 120/72 = 1.67 (95% CI 1.24–2.23), suggesting that PE alone was significantly more common than ED alone.

### 3.3 Association patterns between ED severity and the risk of PE comorbidity

**Figure 1:**
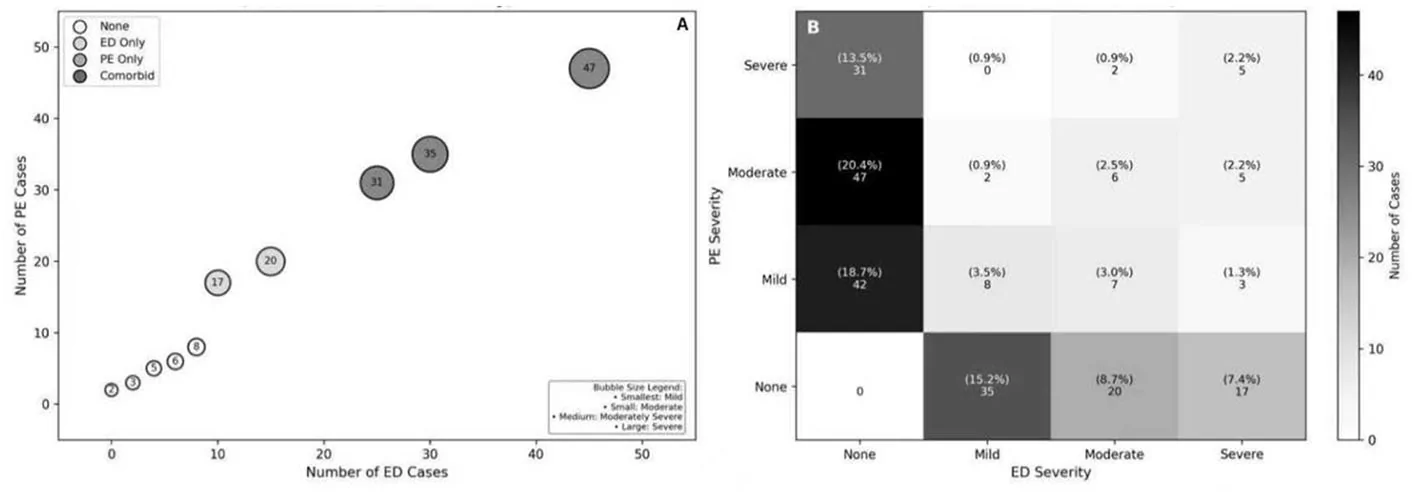
**(A)** A bubble plot showing the distribution of case counts (bubble size indicates severity); **(B)** a heatmap showing the distribution of case counts across different severity combinations.

**Figure 2:**
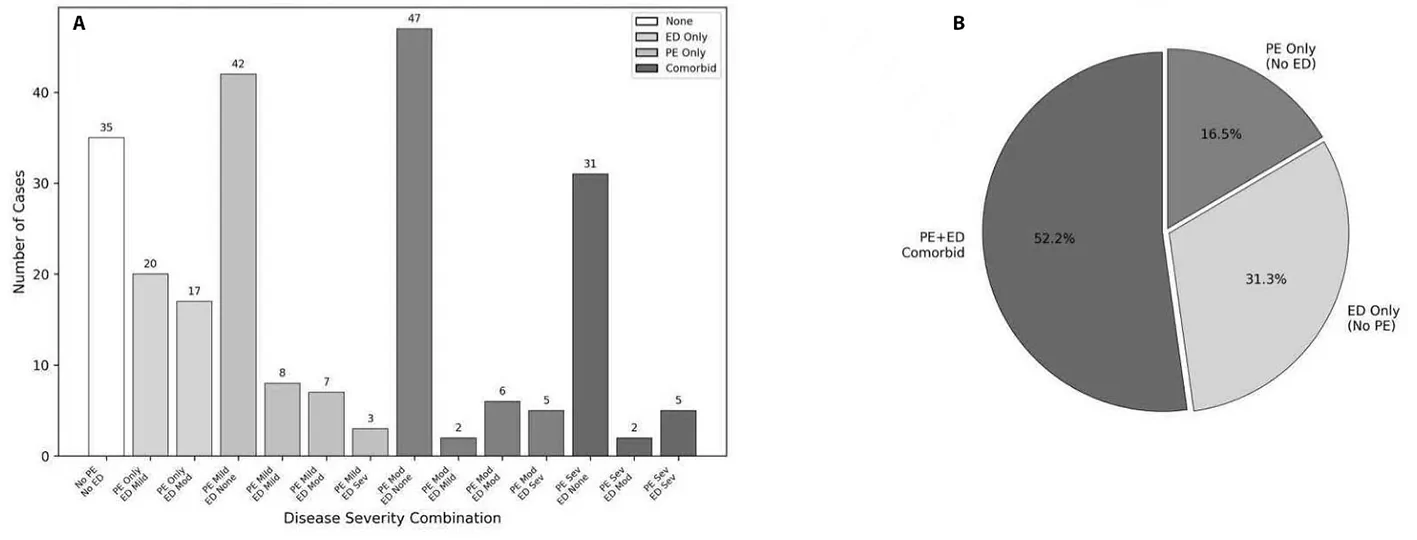
**(A)** A histogram showing the distribution of severity combinations; **(B)** a pie chart showing the distribution of disease patterns (pure premature ejaculation (16.5%), pure erectile dysfunction (31.3%), comorbid conditions (52.2%)).

Figures 1, 2 present an analysis of the relationship between premature ejaculation (PE) and erectile dysfunction (ED) among 230 patients across different severity levels (none, mild, moderate, severe). Each figure consists of two sub-figures.

ED severity showed a unidirectional association with PE risk; comorbidity rates increased from mild ED (*n* = 45; 10/45, 22.2%) to moderate ED (*n* = 35; 15/35, 42.9%) but plateaued thereafter (severe ED: *n* = 30; 13/30, 43.3%). The Cochran-Armitage trend test revealed that, in the study sample, the comorbidity rate of PE showed a significant upward trend as the severity of ED increased (*Z* = 2.31, P = 0.021). Given that observational data show very similar rates of PE comorbidity among patients with moderate and severe ED (42.9% vs. 43.3%), the “proportional odds assumption” underlying ordered logistic regression may not hold. Therefore, we employed multinomial logistic regression for a more detailed analysis. Compared with the mild ED reference group, the risk of PE comorbidity was significantly higher among patients with moderate ED (OR = 2.66, 95% CI: 1.42–4.98, *P* = 0.002); however, the risk of comorbid PE in patients with severe ED did not increase significantly further compared to those with moderate ED (OR = 1.02, 95% CI: 0.62–1.68, *P* = 0.932). A direct comparison between patients with severe ED and those with mild ED showed that the risk of comorbid PE remained significantly elevated (RR = 1.95, 95% CI: 1.02–3.74, *P* = 0.045), but this risk was primarily driven by the difference between mild and moderate severity. To further assess the independent association between ED severity and PE presence, we performed a multivariable binary logistic regression analysis adjusting for age, BMI, and marital status. The adjusted odds ratios for PE presence were: moderate ED vs. mild ED: aOR = 2.47 (95% CI: 1.30–4.69, *P* = 0.006); severe ED vs. mild ED: aOR = 2.52 (95% CI: 1.32–4.81, *P* = 0.005). These results confirm that the association between ED severity and PE risk remains significant after adjustment for potential confounders. To assess the reverse relationship, we performed a multinomial logistic regression analysis with PE severity (mild, moderate, severe) as the independent variable and ED presence (yes/no) as the dependent variable, using the same cohort of 230 patients. This reverse analysis showed that PE severity had no significant effect on ED incidence (χ^2^ = 2.01, df = 2, *P* = 0.367; see Supplementary Table S3 for detailed results), further supporting the asymmetric nature of the association between the two conditions.

In summary, in this cross-sectional sample, we observed a clear statistical association between the severity of ED and the risk of PE. A key feature of this association pattern is non-monotonicity: specifically, the increase in the probability of PE comorbidity primarily occurs during the progression of ED from mild to moderate severity (OR = 2.66), whereas no statistically significant additional increase in risk was observed when ED further worsened from moderate to severe (moderate vs. severe: OR = 1.02). Reverse analysis did not reveal a significant effect of PE severity on the incidence of ED (*P* = 0.367).

A summary of ED severity subgroups is as follows: mild ED (*n* = 45), moderate ED (*n* = 35), and severe ED (*n* = 30). The small sample size in the severe ED subgroup (*n* = 30) should be considered when interpreting the plateau effect, as it may limit the statistical power to detect modest differences between moderate and severe ED groups.

## 4 Discussion

This cross-sectional study examined the comorbidity between ED and PE, focusing on the directionality of their association and the impact of disease severity on comorbidity risk. We observed a marked asymmetry in comorbidity distribution (PE alone: *n* = 120; ED alone: *n* = 72; *P* = 0.0007) and found that PE comorbidity rates increased from mild to moderate ED (22.2% → 42.9%) but plateaued thereafter (43.3%), with a significant difference between severe and mild ED (*P* < 0.05). In contrast, PE severity did not significantly correlate with ED incidence (*P* > 0.05). The comparable sexual activity frequency across groups suggests that these differences reflect genuine condition-related factors rather than reduced sexual exposure. These findings are discussed below in the context of existing literature and potential mechanisms.

These results align with previous reports to a certain extent, further confirming the widespread prevalence of ED and PE comorbidity and the underlying network of shared pathophysiological and psychological mechanisms (14, 18). However, previous studies have primarily focused on describing the prevalence of comorbidity, with limited exploration of potential “directional” differences in comorbidity patterns and the specific role of disease severity (19). This study not only quantified the asymmetry—where isolated PE is significantly more common than isolated ED—but also clarified the key association that “the severity of ED is a unidirectional association pattern of PE risk.” This finding advances the discussion of comorbidity from the question of “whether they coexist” to a more nuanced level of “how and why they are associated,” providing a new starting point for subsequent mechanistic elucidation and clinical practice (20, 21).

The observed unidirectional association may be explained by psychobehavioral and neurobiological mechanisms, but these interpretations require caution. Psychologically, performance anxiety in moderate-to-severe ED may lead to rapid ejaculation (7, 22); external studies support a link between anxiety and PE (23, 24), but we did not measure anxiety. Neurobiologically, shared mechanisms such as endothelial dysfunction, chronic inflammation, and imbalances in the nitric oxide/cGMP and serotonergic pathways have been proposed (14, 25, 26), yet we lacked biomarker data. Notably, the “plateau effect” (42.9% vs. 43.3% from moderate to severe ED) challenges linear models, suggesting unmeasured confounding, risk saturation, or limited power. Therefore, future studies incorporating biomarker measurements, neuroimaging, and longitudinal designs are needed.

Based on these observational findings, ED severity is insufficient as a routine screening indicator for PE pending prospective validation. Nevertheless, clinicians evaluating patients with mild-to-moderate ED should remain vigilant for PE comorbidity and may consider using standardized questionnaires for systematic assessment. Although the PE comorbidity rate was numerically highest in the severe ED group (43.3%), the mild-to-moderate ED group warrants particular attention for several reasons. First, the absolute number of patients with mild-to-moderate ED (*n* = 80) substantially exceeds that of severe ED (*n* = 30) in this cohort, making it a larger target population for screening. Second, the most pronounced increase in PE risk occurs during the transition from mild to moderate ED (22.2% → 4 0.9%; OR = 2.66), whereas no significant additional risk is observed from moderate to severe ED (42.9% → 43.3%; OR = 1.02). This suggests that the critical window for intervention lies in the early stages of ED progression. Therefore, from a public health perspective, prioritizing screening in patients with mild-to-moderate ED captures a larger at-risk population at an earlier, potentially more actionable stage. For confirmed ED-PE comorbidity, sequential management—addressing shared etiologies (e.g., metabolic syndrome, anxiety) followed by pharmacotherapy (e.g., PDE5 inhibitors combined with dapoxetine) combined with psychosexual intervention—may help break the cycle of anxiety and performance failure. However, these recommendations derive from correlational data and require confirmation in prospective interventional studies.

Ultimately, we found no significant association between PE severity and ED incidence, suggesting an asymmetric association pattern in which ED severity is associated with PE risk, whereas the reverse association (PE severity with ED) was not observed. However, due to the cross-sectional design, this interpretation should be regarded as hypothesis-generating rather than confirmatory. This suggests a possible comorbidity framework: ED may represent a more fundamental condition involving multisystem dysregulation (e.g., vascular, neural, endocrine), while PE may be a phenotypic manifestation triggered or exacerbated by it. This “underlying pathology–triggered manifestation” model requires confirmation from future longitudinal studies to demonstrate that changes in ED severity precede changes in PE risk and to identify measurable mediating mechanisms. Until such evidence is obtained, the model should be regarded as a scientific hypothesis awaiting verification.

From a public health standpoint, the identification of a unidirectional association and a ‘plateau effect' provides preliminary, hypothesis-generating insights. However, given the single-center clinical nature of this cohort without a healthy control group, these findings should be considered exploratory and require validation in population-based studies before any broad screening recommendations can be made. First, within this clinical cohort, the findings suggest that PE screening may be prioritized in ED patients, particularly those with mild-to-moderate ED. Second, the findings underscore the need for health education campaigns aimed at reducing performance anxiety, a key modifiable factor that appears to bridge ED and PE. Such interventions, if implemented at the community level, could effectively reduce the dual burden of these conditions, aligning with the goals of promoting mental wellbeing and reducing the burden of non-communicable diseases (*SDG 3*). In conclusion, while our findings highlight a potentially important association pattern in a clinical population, they should be considered exploratory and hypothesis-generating. Population-based studies with longitudinal designs and comprehensive covariate adjustment are needed to determine whether the observed patterns are generalizable to the broader male population and whether they have public health implications.

This study has several limitations. First, the cross-sectional design cannot establish causality or temporality. Any interpretation of directionality is speculative and requires confirmation through prospective longitudinal studies. The associations reported here should therefore be regarded as hypothesis-generating rather than conclusive evidence of a directional relationship. Second, the single-center outpatient sample may introduce selection bias, limiting generalizability. Third, disease diagnosis and severity assessment relied on self-report scales; future studies could incorporate objective measures (e.g., IELT, nocturnal penile tumescence monitoring) to enhance reliability. Fourth, confounding factors such as medication use, partner sexual function, and sociocultural factors were not thoroughly analyzed. Fifth, the absence of a healthy control group precluded estimation of the background prevalence of ED or PE in the general population. Sixth, all participants were men aged 22–45 years presenting with ED and/or PE to a single reproductive medicine center, and no healthy or community-based control group was included. Consequently, the reported prevalence estimates and comorbidity patterns reflect the characteristics of this selected clinical population and should not be extrapolated to the general male population. The public health implications of this study should therefore be interpreted with caution and require validation in population-based cohorts. Additionally, because the data potentially violated the proportional odds assumption of ordered logistic regression, we employed multinomial logistic regression, which confirmed that risk changes were primarily concentrated in the mild-to-moderate ED stage. Future studies analyzing similar ordered exposure variables should pre-test and report the proportional odds assumption. The severe ED subgroup was relatively small (n = 30), limiting the statistical power and precision of estimates for this group. This is particularly relevant for the interpretation of the plateau effect between moderate and severe ED, as the limited sample size may have precluded detection of subtle but potentially meaningful differences.

Future research should focus on: (1) prospective longitudinal studies to clarify causal sequencing; (2) multicenter, community-based surveys with stratified designs; (3) imaging (e.g., fMRI) combined with biomarkers to explore central and peripheral mechanisms; (4) multidisciplinary interventions integrating pharmacotherapy, cognitive behavioral therapy, and couples counseling.

## 5 Conclusion

This cross-sectional study identified an asymmetric comorbidity pattern between ED and PE, with ED severity—particularly the progression from mild to moderate—showing a stronger association with PE risk than the reverse in this cohort. Due to the cross-sectional design, these findings should be interpreted as descriptive associations rather than causal relationships. The findings quantify this asymmetry and, for the first time, report a “plateau effect”: PE comorbidity risk increases significantly from mild to moderate ED, but the additional increase from moderate to severe ED is not statistically significant. This nonlinear relationship warrants further investigation. While PE comorbidity rates were highest in severe ED, the mild-to-moderate ED stage represents a larger target population and the critical window for risk escalation. Clinicians should therefore remain vigilant for PE comorbidity during early ED management, particularly as patients transition from mild to moderate ED.

## Data Availability

The original contributions presented in the study are included in the article/Supplementary material, further inquiries can be directed to the corresponding author.
