# Supplementary material for: The relationship between the severity of erectile dysfunction and the risk of co-occurrence with premature ejaculation: a cross-sectional study
Source: Front Public Health. 2026 Sep 10;14:1956425. doi: 10.3389/fpubh.2026.1956425 (PMC13600813; doi:10.3389/fpubh.2026.1956425)
Supplement: Supplementary file 1 [file Table_1.docx]

**Supplementary Information**

***Table S2. Sensitivity analyses for the association between ED severity and PE risk***

| **Sensitivity analysis** | **Analytical approach** | **Core results** | **Consistency with primary analysis** |
| --- | --- | --- | --- |
| **Analysis 1** | Logistic regression with IIEF-5 as a continuous variable; PE treated as a binary outcome (present vs. absent) | Each 5‑point decrease in IIEF‑5 score (indicating more severe ED) was associated with an increased risk of PE (OR = 1.40, 95% CI: 1.08–1.81, *P* = 0.011) | Consistent |
| **Analysis 2** | Repeating the primary analysis after excluding borderline cases (IIEF‑5 = 21 or CIPE‑5 = 8–9; n = 206 after exclusion) | Mild‑to‑moderate ED: OR = 2.58 (95% CI: 1.35–4.93, *P* = 0.003); Moderate‑to‑severe ED: OR = 1.05 (95% CI: 0.61–1.81, *P* = 0.856) | Consistent (the nonlinear “plateau effect” remained) |

*Notes:* The primary analysis revealed a nonlinear association between ED severity and PE risk (mild‑to‑moderate: OR = 2.66; moderate‑to‑severe: OR = 1.02). Both sensitivity analyses yielded results consistent in direction and magnitude with the primary findings, supporting the robustness of the main conclusions. For Analysis 1, a 5‑point decrease in IIEF‑5 score corresponds approximately to one severity grade increment, and PE was analyzed as a binary outcome (presence vs. absence). Borderline cases in Analysis 2 were defined as IIEF‑5 = 21 (threshold for ED diagnosis) or CIPE‑5 = 8–9 (threshold between moderate and severe PE); after exclusion, the sample size was 206, with group sizes: PE only (n = 106), ED only (n = 62), and comorbid (n = 38). The CIPE‑5 total score ranges from 0 to 20, with 4–8 indicating severe PE, 9–12 moderate PE, 13–16 mild PE, and 17–20 no PE. All analyses were performed using the same multinomial logistic regression model as the primary analysis, with PE severity as the ordered categorical outcome.

***Table S3. Reverse analysis: association between PE severity and ED incidence (multinomial logistic regression)***

| **Independent Variable** | **n** | **ED incidence, n/N (%)** | **OR (95% CI)** | **P-value** |
| --- | --- | --- | --- | --- |
| **PE Severity (mild)** | 60 | 18/60 (30.0) | Reference | — |
| **PE Severity (moderate)** | 60 | 13/60 (21.7) | 0.65 (0.29–1.45) | 0.293 |
| **PE Severity (severe)** | 38 | 7/38 (18.4) | 0.53 (0.21–1.33) | 0.177 |

*Notes:* Multinomial logistic regression was performed with PE severity as the independent variable (mild PE as reference) and ED presence as the dependent variable. Overall model: χ² = 2.01, df = 2, P = 0.367. Data are presented as odds ratios (OR) with 95% confidence intervals (CI). The small sample sizes in the PE severity subgroups (mild: n=60; moderate: n=60; severe: n=38) should be considered when interpreting these results. The overall model P = 0.367 indicates no statistically significant association between PE severity and ED incidence, consistent with the primary finding that the association is unidirectional.
